# Modeling of Impurities Evaporation Reaction Order in Aluminum Alloys by the Parametric Fitting of the Logistic Function

**DOI:** 10.3390/ma17030728

**Published:** 2024-02-03

**Authors:** Aleksandar M. Mitrašinović, Jasmina Nešković, Svetlana Polavder, Sandra Petković, Željko Praštalo, Nebojša Labus, Milinko Radosavljević

**Affiliations:** 1 The Department of Materials Science and Engineering, University of Toronto, Toronto, ON M5S 3E4, Canada; 2 Institute of Technical Sciences of the Serbian Academy of Sciences and Arts, 11000 Belgrade, Serbia; 3 Mining Institute Belgrade, 11080 Belgrade, Serbia

**Keywords:** evaporation, high temperature, pressure, aluminum, alloy, logistic function, modeling

## Abstract

Advancements in computer capabilities enable predicting process outcomes that earlier could only be assessed after post-process analyses. In aerospace and automotive industries it is important to predict parts properties before their formation from liquid alloys. In this work, the logistic function was used to predict the evaporation rates of the most detrimental impurities, if the temperature of the liquid aluminum alloy was known. Then, parameters of the logistic function were used to determine the transition points where the reaction order was changing. Samples were heated to 610 °C, 660 °C, 710 °C, and 760 °C for one hour, after which the chemical analyses were performed and evaporation rates were calculated for Cd, Hg, Pb and Zn elements. The pressure inside the encapsulated area was maintained at 0.97 kPa. Whereas parameters that define the evaporation rate increase with the temperature increase, the maximum evaporation rates were deduced from the experimental data and fitted into the logistic function. The elemental evaporation in liquid-aluminum alloys is the best defined by the logistic function, since transitions from the first to zero-order-governed evaporation reactions have nonsymmetrical evaporation rate slopes between the lowest and the highest evaporation rate point.

## 1. Introduction

The demand for aluminum and its alloys formed from the liquid state is expected to grow significantly in the following decades. Their lightweight properties, durability, corrosion resistance, recyclability and good mechanical properties expand the scope of application of the aluminum alloys from the predominantly aerospace and automotive industries to communications and electronics, as well as to agricultural, construction, and mining equipment, and other parts of heavy industry and machinery that traditionally uses iron-based materials [1]. The global market size of aluminum and its alloys formed from the liquid state was estimated at USD 86.92 billion in 2021 and is expected to exceed USD 100 billion in 2026 [2]. With the widening range of applications, the demand for high-quality aluminum products has increased, which consequently opens the need for new mathematical modeling and quality control tools.

**Figure 1:**
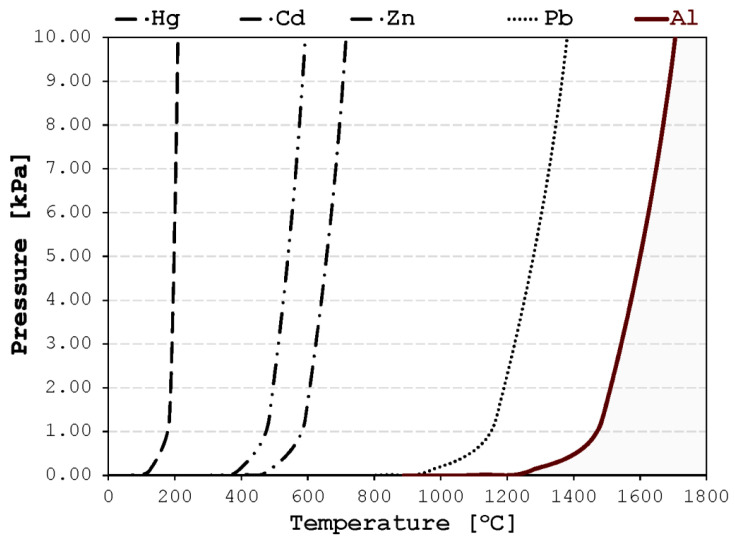
Vapor pressure change of Al, Cd, Hg, Pb, and Zn with the temperature increased. Diagram data extrapolated from the HSC Chemistry 5 Thermochemical Calculation Software, v5.0.

The properties of the aluminum alloys are well examined topics where Cd, Hg, and Pb are often noted as the most unwanted metallic elements [3,4]. In recent years, with the advances in additive manufacturing, Zn is used in aerospace and defense industries to improve the corrosion resistance of aluminum alloys [5], although Zn is traditionally considered an impurity element due to the ease of microsegregation [6]. The presence of these elements causes a lower reliability of the solidification process and a reduction in mechanical properties in final products [7]. The removal, or at least control, of unwanted elements from the aluminum alloys is an important processing step in the metal casting industry [8,9]. Liquid aluminum alloys are subjected to a low-pressure environment primarily to remove dissolved hydrogen and other gases [10,11] or for making a finer microstructure [12]. However, low-pressure treatment of liquid aluminum alloys reduces the amounts of all the elements with higher vapor partial pressure values than aluminum [13]. Since the partial pressures of most unwanted metallic elements are above the values of the vapor partial pressure of aluminum itself, the vacuum refining process is the most direct way to remove a significant amount of these contamination components from liquid aluminum. Therefore, if the aluminum alloy is heated up at a temperature above its melting point and kept at low pressure for a substantial time, most of the unwanted elements will evaporate from liquid alloy at a higher rate than aluminum (Figure 1).

The elemental evaporation of each element is governed by the value of the vapor pressure of a particular element above the liquid alloy [14]. If the temperature of the liquid alloy is above temperature where the elemental vapor pressure is equal to the ambient pressure, the particular element will readily evaporate. Although each evaporating element has a specific value and rate of change, the vapor pressure of all elements significantly changes with temperature [15]. The longer the liquid alloy is kept at a high temperature and the greater the degree of vacuum, then the greater the rate of elemental evaporation. The Hertz–Knudsen equation describes how substances evaporate in vacuum or conditions close to it. It shows that the temperature at the interface (*T*), molar mass (*M*), standard vapor pressure (*p*^0^) of the element, and pressure above the liquid bath (*p*) are all directly correlated with the maximum molar flux of evaporation of that element.

(1) $${ή}_{E}=\frac{p^{0}-p}{\sqrt{2\pi MRT}}$$

In complex alloys subjected to high temperatures and low pressure, more phenomena than only evaporation occur that would divert effective evaporation rates from theoretically calculated values, e.g., by decreasing the distance to the free surface, dissolved gases can greatly increase evaporation ratios, or some elements can react among themselves, while others in combination with gas bubbles can act as nucleation sites, and form different compounds inside liquid alloy. Fang and Ward [16] were one of the first to recognize discrepancies between temperature and expected evaporation rates. Hoyst et al. [17] used molecular dynamics simulations on a variety of thermodynamic settings to find more than three times larger evaporation changes than would be predicted by the Hertz–Knudsen equation. In practice, due to the complexity of the secondary-aluminum alloys and uncertainties of initial conditions and process parameters, the experimental findings diverge from theoretical evaluations.

In order to assess the evaporation rate in batch-type thermodynamically equilibrated systems intended for high-temperature low-pressure aluminum alloy refining, this work considers a representative mathematical correlation between initial chemical composition and the alloy’s holding temperature. Parameters that define the evaporation rate increase with the temperature increase and maximum evaporation rate was correlated to the components of the logistic function. The developed logistic function is validated through comparison with the experimental results. A new model is further used to identify the transitional temperatures for evaporation reaction-order change.

## 2. Refining Aluminum at Low Pressure

Aluminum can have different elements added to it to enhance its machinability, malleability, ductility, tensile strength, hardness, corrosion resistance, etc. Aluminum–copper alloys may benefit from the addition of cadmium, an element with a relatively low melting temperature that accelerates age hardening and improves strength and corrosion resistance. Cd, at amounts of 0.005 to 0.5%, is used to shorten the period that alloys made of aluminum, zinc, and magnesium age. Lead is incorporated into certain alloys at approximately a concentration of 0.5% to improve machinability. Mercury is utilized at a concentration of 0.05% in sacrificial anodes for the protection of steel structures. Besides its intended purpose, the introduction of Hg into aluminum, regardless of whether it is in the form of a metal or a salt, will trigger accelerated corrosion in the majority of aluminum alloys. The addition of other elements in aluminum–zinc alloys yield the most optimal combination of tensile properties in wrought-aluminum alloys. Nevertheless, the casting alloys with the zinc in it are susceptible to hot cracking, and the wrought alloys are prone to stress-corrosion cracking.

In order to reuse the secondary alloys, it is necessary to eliminate certain undesirable elements. The presence of elements with a relatively low melting point in secondary-aluminum alloys significantly impairs the fatigue resistance of the alloys. These constituent elements give rise to phases that typically solidify late, resulting in their formation at grain boundaries and interdendritic regions, which diminish the mechanical properties of the aluminum alloys. Additionally, the majority of intermetallic phases have a tendency to form sharply edged platelets and are extremely brittle; as a result, they can serve as low-energy pathways or accentuate raisers for the nucleation or propagation of cracks [18].

In secondary steelmaking, high-temperature refining principles under low vacuum have long been used for degassing and decarburization. Thermodynamic parameters published by Bauer et al. [19] lead to vacuum refining procedures that remove undesired carbon with little loss of chromium and other alloying components. Emphasizing the vacuum degassing, Capurro et al. [20] found a 60% decrease in inclusion density in liquid steel. Similar results were seen by Mitrašinović et al. [21] in silicon following treatment at temperatures of around 1600 °C and pressures of fewer than five kPa, where inclusions settled as a fine film at the crucible’s top edge and the bulk concentrations of trace elements were reduced several times. Jia et al. [22] used a vacuum distillation technique to successfully remove lead and tin from soldering materials. The vacuum induction melting processes are beginning to be increasingly employed for the synthesis of high entropy alloys [23]. Within the liquid aluminum processing sector, dissolved hydrogen and other gases are eliminated by maintaining liquid alloy at low pressures. Simultaneously, similarly to the dissolved gases, all the components in the liquid alloy evaporate at the same degree during the low-pressure treatment. For assessing porosity in Al-Si eutectic alloys, Kumar and Sundarraj [24] proposed combining the foaming approach with low pressure. They also documented alterations in the chemical composition of specific non-alloying components. Following that, Mitrašinović and D’Souza [25] verified that the Al7Si4Cu alloy underwent a discernible change in chemical composition when it cooled from 760 °C at 2.1 kPa, despite that they only created low pressure during the solidification phase and at comparatively low temperatures. Thus, the concentration drop of various harmful elements from the aluminum alloy held in vacuum appears to be a viable assumption.

Monitoring process parameters at the high temperatures in a pressure-controlled environment is challenging and typically relies on a limited number of experimental data. The current study aims to compare actual results with the mathematical model that establishes the evaporation parameters of the main impurities in the molten secondary-aluminum alloy kept in low vacuum. Using the determined kinetics parameters, the susceptibility of elements to evaporation at different temperatures was measured. The logistic function’s parameters, transition points, elemental evaporation ratios, and reaction orders were all inferred from experiments conducted at temperatures ranging from 610 to 760 °C.

## 3. Materials and Method

**Figure 2:**
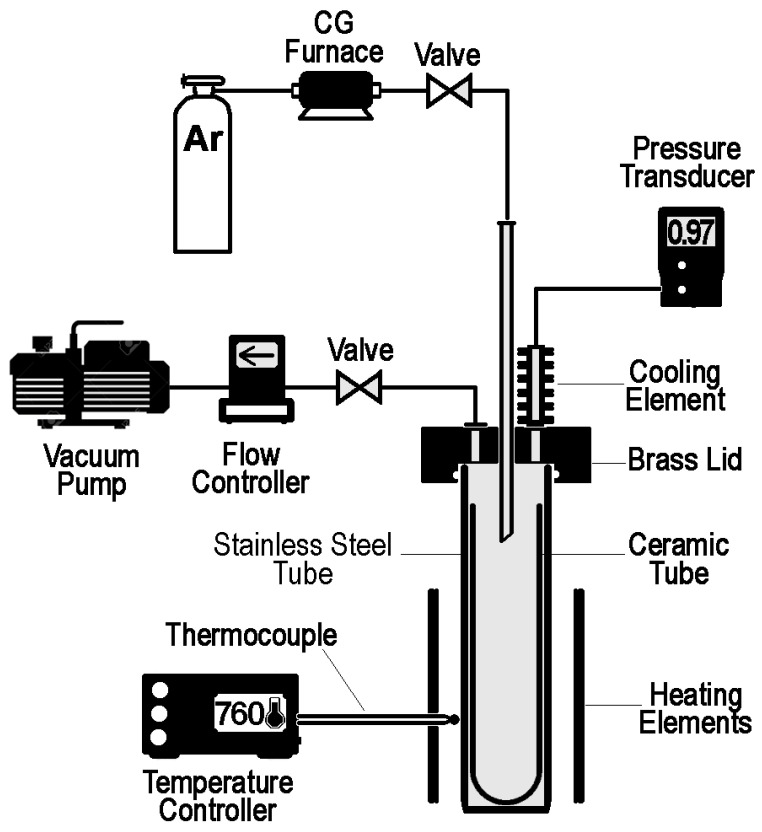
Delineation of the test apparatus.

A stainless steel closed-end tube was rested inside an electro-resistant furnace, as shown in Figure 2. An alumina closed-end tube with a 5.08 cm inner diameter was fitted inside the stainless tube. The alumina tube’s bottom was filled with 200 g of the aluminum alloy piece. The opposite end of the stainless steel tube was then sealed with two of the O-rings that could withstand high temperatures, enclosed by a brass cap. Prior to heating, a high-purity argon gas was injected into the encapsulated area to remove oxygen and other possibly chemically active gases and flashed through the copper getter chamber to eliminate any remaining moisture.

**Figure 3:**
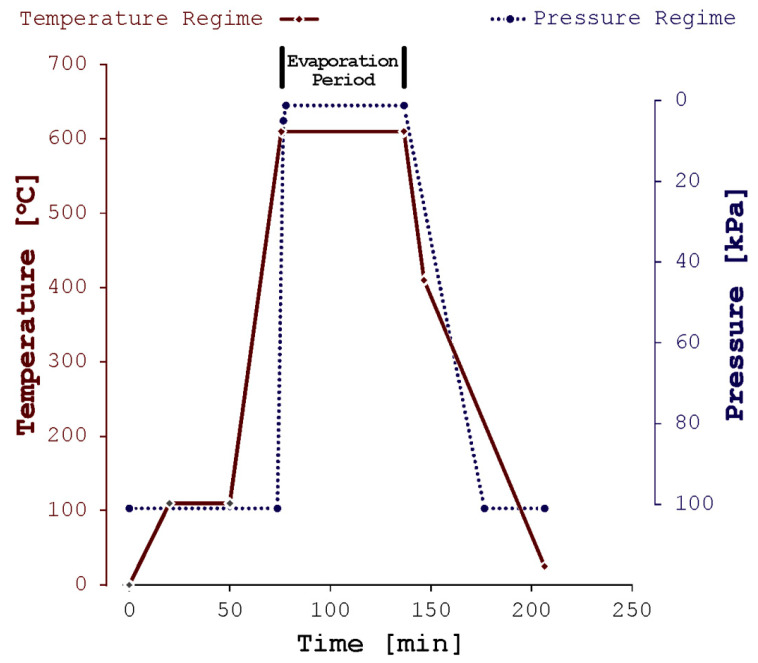
Temperature and pressure regime for experiment conducted at 610 °C. Note that the alloy melting point (liquidus) is at 597.1 °C while solidus temperature is at 460.4 °C.

The rapid heating was supplied by the molybdenum–disilicide elements. The temperature control was performed with the B-type thermocouple. The schematics of the operational parameters are given in Figure 3. To remove traces of humidity from the experimental system, the furnace was heated slowly to 110 °C and was left for 30 min at that temperature. From 110 °C to the set temperature, the furnace was heated at a rapid rate, 32 °C per minute. One minute before reaching the set temperature, a vacuum pump was turned on. To protect the refractory vessel, pressure was gradually reduced from 101 kPa to approximately 5 kPa in three minutes. Once the pressure achieved 5 kPa, a vacuum pump was set at maximum capacity to achieve a maximum vacuum level of 0.97 kPa. After one hour, a stainless-steel tube was removed from the furnace and left to cool down to room temperature. 

In all instances, the pressure inside the encapsulated area was maintained at 0.97 kPa. Table 1 provides the chemical composition of the starting aluminum alloy. A thermal analysis approach was employed for determining the beginning of the melting of the aluminum alloy, which was 597.1 °C [26]. The ratio of the free evaporating surface to the volume of the liquid aluminum alloy was 1.28 m^−1^ in all instances, since the inner diameter of the vessel was 5.08 cm and the weight of the liquid alloy was 200 g. Temperature homogeneity, alloy homogeneity in liquid state, and the effect of the area of evaporation surface are described in the work of Mitrašinović and Odanović [27].

Later, the alumina tube was taken out from the stainless-steel tube and then the aluminum alloy was separated from the alumina tube. Then, gravimetric analyses were completed. In order to obtain the average chemical composition of the entire sample after gravimetric analyses, samples were crushed to pieces smaller than 2000 microns and stirred until uniformity was reached. Two grams of particles of each sample were collected and sent for chemical analyses. All chemical analyses were obtained by the inductively coupled plasma mass spectrometry analyses in the International Plasma Laboratory (Richmond, BC). Table 2 shows the concentrations of the Cd, Hg, Pb, and Zn in the initial aluminum alloy and after one hour treatment at low pressure.

## 4. Gravimetric Analysis

**Figure 4:**
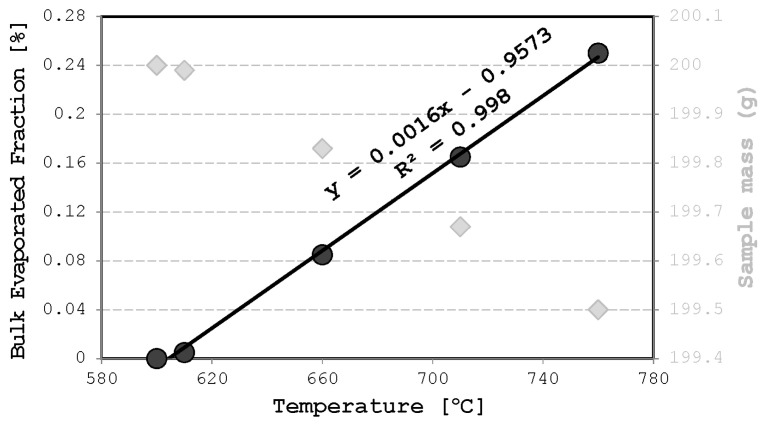
The correlation between specimens’ mass loss due to evaporation and holding temperature of the liquid alloy.

Gravimetric analysis, given in Figure 4, showed that along with the evaporation of impurities, a recognizable evaporation of bulk elements may occur. In the current investigation, the losses between 0.005 mass percent at a temperature of 610 °C and 0.25 mass percent losses at 760 °C are measured. At temperatures below 760 °C, the excessive evaporation of bulk material does not begin, since the linear equation describing the correlation between overall specimens’ mass loss due to evaporation and the holding temperature of the liquid alloy has a coefficient of determination 0.998. 

## 5. Utilization of the Logistic Function

From the lowest to the greatest points of transformation rate, the logistic function has nonsymmetrical slopes and describes natural occurrences with forward and backward transitions from first- or second-order chemical reactions to zero-order chemical reactions. Thus, it is commonly used in biological [28,29], genetic engineering [30], and other applied sciences to describe specific phenomena. The application of the logistic function in formalizing chemical reactions that occur at high temperatures and inside encapsulated space [31] is rarely used, mainly due to challenges related to acquiring the required number of experimental probes for accurate experimental-modeled fitting.

The logistic–function curve should ideally match the real values, accurately depicting the concentration vs. response relationship free from error divergences. The resulting curve would be the true curve if an unlimited number of measurements were made, each with an infinite number of reproductions. Practical constraints limit the number of samples that can be run, making it especially challenging to run numerous samples in a confined environment at high temperatures. As a result, the correct curve must be calculated from a few experimental findings. In order to construct the logistic function, at least five parameters must be obtained from the experiments. Here, evaporation rates were obtained from the chemical compositions taken in samples before and after vacuum treatment. Therefore, five parameters were the evaporation rates obtained at the four temperatures 610, 660, 710, and 760 °C, while the fifth point was deduced at the liquidus temperature at 597.1 °C. 

**Figure 5:**
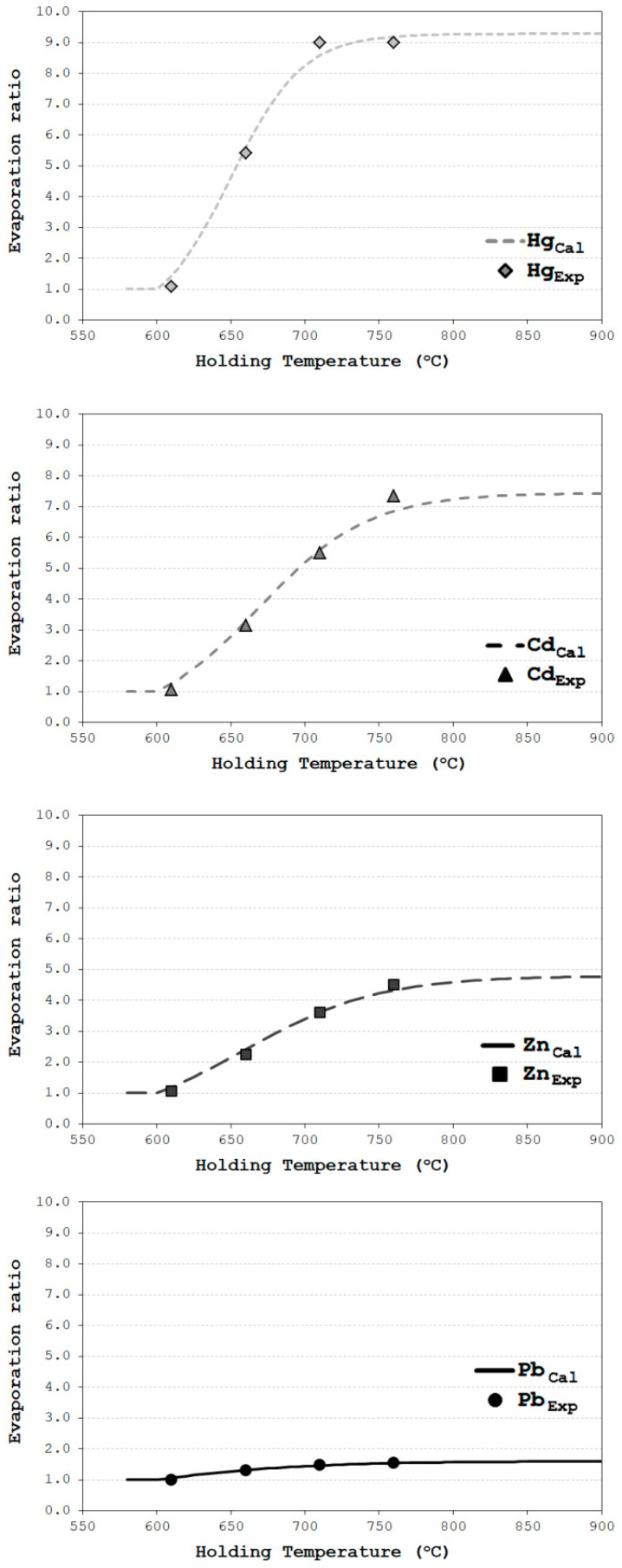
The calculated logistic–regression curves for four impurity elements together with corresponding experimentally obtained results.

In this work, a unitless evaporation rate ratio between the concentrations of a particular element before and after the low-pressure treatment at a particular temperature was obtained from the experimental measurements (Table 2). Elemental evaporation from liquid aluminum alloys, for temperature increase, changes its reaction order from first order to zero-order reaction and therefore the most adequate mathematical form that describes such a process is the logistic function. Logistics function, in this particular case, provides control of two mathematical functions that correspond to two key process parameters that are, precisely, elemental evaporation reaction rate increase for temperatures where the first-order reaction is predominant and maximum evaporation rate at high temperatures where reactions kinetics are governed by zero-order reactions. The general form of the logistic function is defined by the following equation:

(2) $$f\left(x\right)=\frac{1}{1+e^{-t}}\equiv \frac{1}{1+exp\left(-t\right)}$$

that can be rewritten as:

(3) $$E_{cal}=\frac{1}{1+e^{-\tau }}$$

where *E_cal_* is the calculated evaporation rate and *τ* is the absolute temperature corresponding to the temperature at which liquid alloy is held. Equation (3) can be formalized by the following differential equation:

(4) $$\frac{dE_{cal}}{dt}=\alpha E_{cal}\left(1-\frac{E_{cal}}{E_{max}}\right)$$

where α represents the slope on the curve where evaporation is governed by the first-order chemical reactions and the evaporated fraction increases if the holding temperature is higher. The maximum capacity of the vacuum treatment is expressed by the value of *E_max_* where the evaporation is governed by the zero-order chemical reaction distinctive by a constant value for the evaporation reduction factor regardless of the temperature increase. Thus, the solution for the differential equation for elemental evaporation rate for temperature is:

(5) $$E_{cal}=\frac{E_{max}E_{initial}e^{\alpha \tau }}{E_{max}{+E}_{initial}\left(e^{\alpha \tau }-1\right)}$$

where *E_initial_* is the initial value of the element’s evaporation rate, which is always equal to one because in the aluminum alloys’ solid structure evaporation does not occur. The absolute term for temperature τ can be replaced with the temperature difference value between the alloy’s melting point and the temperature at which evaporation occurs. The final form of the logistic function describing the correlation between the evaporated fraction of a particular impurity element in aluminum alloy kept at a particular temperature is:

(6) $$E_{cal}=\frac{E_{max}E_{initial}e^{\alpha \Delta T}}{E_{max}{+E}_{initial}\left(e^{\alpha \Delta T}-1\right)}$$

where Δ*T* is the difference between the actual alloy’s temperature (*T_initial_*) and the lowest temperature (*T_liquidus_*) at which the alloy is liquid. The calculated logistic–regression curves for four impurity elements are given in Figure 5, together with corresponding experimentally obtained results.

Parametric fitting of the constants *α* and *E_max_* with experimental results is completed by using a ‘minimization of a sum of the least squares’ method [32]. By using this method, the sum of the squares of the function’s differences is minimized. The generalized reduction gradient approach (e.g., the nonlinear optimization procedure included in the Microsoft Excel Solver add-in) is used to determine the constants of the logistic function. While it is assumed that up to the temperature of 597.1 °C the chemical composition of the aluminum alloy did not change [25], results at temperatures of 610 °C, 660 °C, 710 °C, and 760 °C were utilized in the analysis of experimental data to optimize the computed evaporation rate values. Minimized square values, given in Table 3, are calculated as:

(7) $${sq}^{\left(\tau \right)}={\left(E_{cal}^{\left(\tau \right)}-E_{exp}^{\left(\tau \right)}\right)}^{2}$$

where $E_{exp}^{\tau }$ is the experimentally obtained value for the evaporation rate at a particular holding temperature (Figure 5). Relatively low minimized square values indicate satisfactory fitting between experimental and calculated values. In process control engineering, the eventual occurrence of larger values of sq would be a valuable tool as an indicator of change in process conditions.

Table 4 shows regression coefficients for distinct elements used to evaluate the logistic function outcomes with the experimental results. The closest correlation between experimental results and calculated values is found for Zn, followed by Cd, and Pb while Hg had noticeable lesser value for regression coefficient.

## 6. Determination of Transitional Points

**Figure 6:**
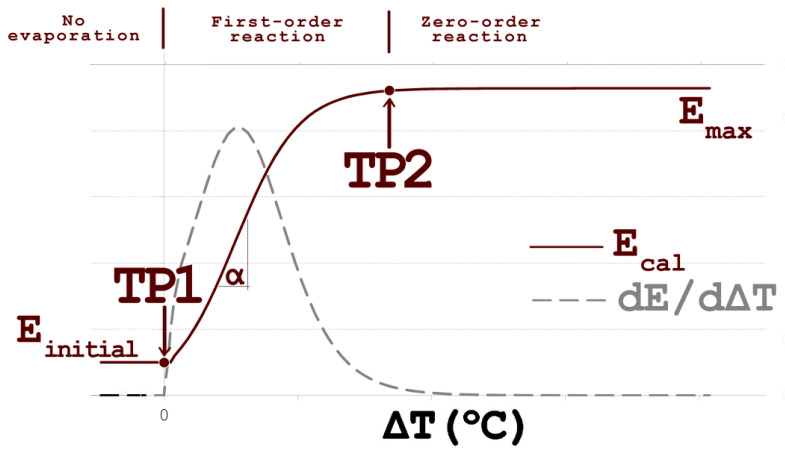
Graphical interpretation of the experimental and calculated parameters used to correlate a change in evaporation rate for different holding temperatures.

Two major transition points characterize the elemental evaporation from liquid alloys at low pressures. The first transition point (TP1) is at the alloy’s liquidus temperature, where the first liquid phase occurs. At high temperatures, the increase of temperature evaporation is governed by the mass transport of dissolved elements to the surface, and therefore evaporation rate is constant. The temperature at which the evaporation rate becomes constant regardless of the temperature is the second transitional point (TP2). The second transitional point corresponds to the change in the evaporation mechanism that in kinetics considerations is the transition point between the first and the zero-order evaporation reaction with the controlling of the temperature of the aluminum liquefied bath. It is possible to determine the transition points’ values from the first derivative of the logistic function, as depicted in Figure 6. The elemental evaporation ratio curve (E) for high temperatures has an asymptotic approach to the value of the maximum capacity of the vacuum treatment (*E_max_*) that could be expressed as:

(8) $$\underset{T\to \propto }{\lim}E_{cal}\left(T\right)=E_{max}$$

To simplify practical applications, the 95% difference method is chosen to delineate the transition point where the evaporation process at a particular holding temperature has a transition in the evaporation mechanism [33]. Thus, the temperature value where the dE/dΔT value is 95% of the maximum value of the dE/dΔT is the transitional temperature, depicted in Figure 6 and given in Table 5 as the TP2 value. 

**Figure 7:**
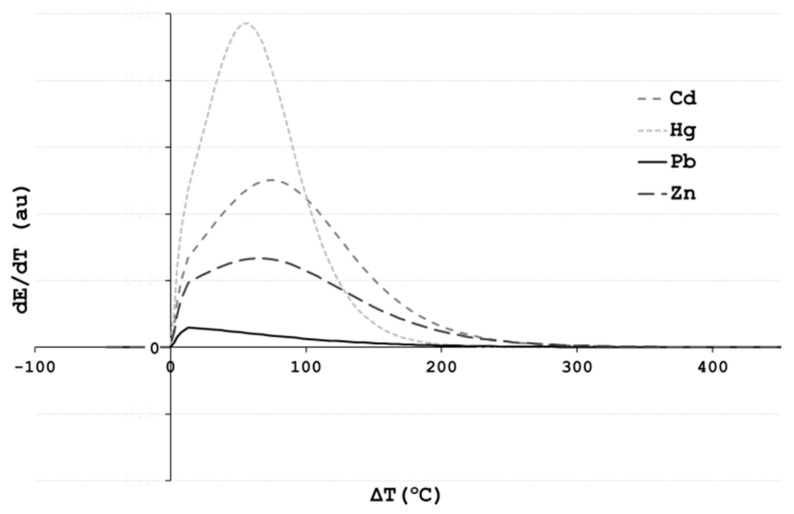
Determination of the transitional points from the first derivative curve of the elemental evaporation rate regarding temperature.

While the beginning of the elemental evaporation is expected to start at the temperature when aluminum alloy becomes liquid, the second transitional point varies greatly for different elements (Figure 7). The second transitional point (TP2) corresponds to the lowest temperature, where vacuum refining treatment reaches its maximum efficiency. Regarding process control parameters, the second transitional point shows the recommended process temperature, where the maximum evaporation occurs at the minimum energy input.

## 7. Discussion

Rapid and accurate control of process parameters is important in practical applications. To maintain consistent quality of solidified components, typical casting plant quality control techniques are a reduced pressure test for the detection of dissolved gases, optical microscopy for microstructure assessment, emission spectroscopy for chemical analysis, or differential thermal analysis for thermochemical characterization [18]. Most producers involved in liquid metal processing already incorporate automated temperature control that requires easy-to-use thermocouples as sensors and computers as data analyzers. However, these resources are often used only to register process parameters. With further improvements in the understanding of the solidification processes and implementation of the new mathematical models for assessment of the process parameters, prediction of the materials’ properties before entering into the production cycle is becoming possible. This work requires the known chemical composition of the initial material and the temperature at which the material will be treated to assess the level of most detrimental impurities in the final parts. Tight monitoring of the production process and prevention of the intrusion of unwanted elements in the production cycle generates higher quality products with longer service time and easier recycling after the end of their life cycle.

### 7.1. Correlation between the Experimental and Thermodynamic Data

Recently, aluminum from secondary sources finds its usage even in applications where high cleanliness of the melt is important. Removing unwanted elements from the aluminum secondary stock is a challenge. The majority of products formed from the liquid aluminum contain significant amounts of alloying elements. Cu, Fe, Mn, Mg, Si, and recently Zn are regularly injected into aluminum to improve the specific properties of the final products. In recent years, the concentrations of the alloying elements in aluminum alloys are increasing, whereas in some cases the concentrations of alloying elements in automotive and aerospace applications formed from liquid state surpass 28% [34]. The ease of removing unwanted elements from the secondary sources by evaporation is a complex process, that in thermodynamics terms is dependent on a large number of parameters, e.g., chemical composition, overall pressure, partial pressures of all the species present in melt, activity coefficients of species with significant concentrations, melt and surrounding temperatures, and activity coefficients of the oxygen and oxidation products. By forming slags under vacuum conditions, elements which exhibit a small activity coefficient of corresponding oxides can be removed in vacuum [35]. Considering the kinetics parameters, once the evaporation process instigates, the vapor pressure of the alloying and unwanted elements will increase until the vapor pressure of these elements and aluminum becomes even, and in that moment, the removal of the unwanted elements turns out to be inefficient. In practical applications, the evaporation of the unwanted elements is additionally contrived by technological specifics that influence the temperature distribution in the holding vessel, change in viscosity and fluidity of liquid metal, the effect of refractory material on liquid metal, and many other parameters. Since removing unwanted elements from the liquid aluminum by vacuum refining is affected by numerous parameters, the outcomes may significantly differ from the theoretical considerations. In that matter, the mathematical modeling of the elemental evaporation from the aluminum is a starting tool to assess the level of unwanted elements in the final product and the base for in situ monitoring by further adopting neural networks programs and in the future independent change in process parameters adopted by artificial intelligence.

### 7.2. Correlation between Alloy’s Chemical Composition and Regression Analyses for Artificial Neural Network Techniques

Most alloying elements are added to aluminum to improve its mechanical properties, e.g., elongation, yield stress, ultimate tensile stress and other. These properties can be controlled by the solidification process parameters or by the heat treatment after forming the final parts. Although extensive research is completed in this field, because of the large number of affecting parameters, the universal theoretical model to predict the mechanical properties thus far has yet to be delivered. The extensive study is being undertaken by the NSERC group in Canada regarding the development of the artificial neural network (ANN) based on multiple regression models to predict the mechanical properties of the A356 alloy from the processing variables. They developed several standard nonlinear regressions and trained multi-layer ANN models using data from the literature and experimental results. The results implied that the usage of the ANN provides far better anticipation of the mechanical properties of the aluminum alloys if only the chemical composition of the starting material and processing variables are available [36].

## 8. Conclusions

In this work, the logistic function is used to predict the evaporation rates of the most detrimental impurities based on the known temperature of liquid aluminum alloy. The five determinants for the logistic function were liquidus temperature of the aluminum alloy and four holding temperatures for the liquid melt, whereas corresponding parameters were acquired from the experimental results. While the beginning of the evaporation begins at the liquidus temperature of the aluminum alloy, the transition temperatures where the mechanism of the evaporation reaction changes from the first order to zero order are found to be 835, 760, 725, and 860 °C for Cd, Hg, Pb, and Zn, respectively. The highest evaporation rate, corresponding to the slope of the first order evaporation reaction, is found to be for Pb, followed by Cd and Zn, while Hg had significantly lesser evaporation rate. Validation of the developed model showed high values for regression coefficients ranging from 0.989 for Zn to 0.847 for Hg. By using developed logistic regression, a single equation can be used for any range of required temperatures in a mathematical model assessing the evaporation process at the high-temperature and low-pressure refining of secondary-aluminum alloys in liquid state and further serves as a base for the development of the neural network’s models. 

## Figures and Tables

**Table 1:** The chemical composition of the initial aluminum alloy. All values are in wt%.

Element	Si	Cu	Sn	Fe	Mn	Mg	Ti	Pb	Ni	Hg	Cd	Zn	Al
Concentration	7.89	3.23	0.4	0.37	0.25	0.17	0.12	0.04	0.04	0.027	0.022	0.018	Rest

**Table 2:** Concentration of the Cd, Hg, Pb, and Zn in initial aluminum alloy and concentrations after one hour treatment at 0.97 kPa pressure. All values are in ppm_wt_.

Element	Measurement Sensitivity	Initial Composition	Composition after the Treatment			
		(below 597.1 °C)	(610 °C)	(660 °C)	(710 °C)	(760 °C)
Cd	10	220	210	70	40	30
Hg	5	270	250	50	30	30
Pb	10	400	400	310	270	260
Zn	10	180	170	80	50	40

**Table 3:** Summarized data for the minimized squares values for differences between calculated and experimental values for four temperatures (sq).

Element	sq^(610)^	sq^(660)^	sq^(710)^	sq^(760)^	sq^(total)^
Cd	0.044	0.016	0.0085	0.23	0.2985
Hg	0.13	0.030	0.18	0.040	0.3800
Pb	0.00	0.00043	0.00044	0.00	0.00087
Zn	0.017	0.023	0.00015	0.298	0.3381

**Table 4:** Regression coefficients (R^2^) for linear fitting (y = ax + b) for calculated and experimentally obtained results.

Element	a	b	R^2^
Cd	0.495	0.480	0.985
Hg	0.644	0.129	0.847
Pb	0.615	0.403	0.966
Zn	0.523	0.488	0.989

**Table 5:** Summarized data for a maximum capacity of evaporation treatment (*E_Max_*), evaporation rate (*α*), and corresponding temperatures for transitional points (TP1 and TP2).

Element	E_initial_	E_max_	α	TP1, °C	TP2, °C
Cd	1	7.44	0.0270	597.1	835
Hg	1	1.60	0.0167	597.1	760
Pb	1	9.28	0.0420	597.1	725
Zn	1	4.79	0.0223	597.1	860

## Data Availability

Data are contained within the article.
